# A Fuzzy Comprehensive CS-SVR Model-based health status evaluation of radar

**DOI:** 10.1371/journal.pone.0213833

**Published:** 2019-03-18

**Authors:** Yifei Yang, Maohui Zhang, Yuewei Dai

**Affiliations:** 1 School of Automation, Nanjing University of Science and Technology, Nanjing, Jiangsu, China; 2 School of Electronics and Information, Jiangsu University of Science and Technology, Zhenjiang, Jiangsu, China; Northeast Electric Power University, CHINA

## Abstract

The purpose of Fuzzy Comprehensive CS-SVR Model (FCCS-SVR) is to evaluate and monitor the health status of a radar equipment and then keep its safe operation. Due to reasons such as few samples, slow changes and the nonlinear structure of data of fault monitoring signal, the health status evaluation of a radar system is quite difficult. By establishing the evaluation index system of a radar, the combination of AHP method and Entropy weight method is studied in this paper. In order to evaluate the value of health status, several optimization algorithms including PSO, GA, BA and CS are used for optimizing the parameters of SVR model. Meanwhile, in order to avoid the problem that the system is at the edge of the state, a radar health assessment method based on the combination of Fuzzy Comprehensive Evaluation and Cuckoo Search-Support Vector Regression (CS-SVR), which is named as Fuzzy Comprehensive CS-SVR (FCCS-SVR), is further proposed. The result of case analysis reflects that the state evaluation of the radar system is realized. The system performance analysis shows that the use of FCCS-SVR evaluation method provides a high recognition rate and can accurately assess the health status of the radar system.

## 1. Introduction

Radar, also known as “radio detection and ranging”, is the use of radio to find targets and determine their spatial position. It is an electronic device that uses electromagnetic waves to detect targets. Therefore, the radar is also called “radio positioning”. According to the applications of radar, the radar equipment includes early warning radar, search and alert radar, altimetry radar, airborne radar, meteorological radar, navigation radar, etc. Various types of radars have now been fully integrated and brought more convenience to human’s life. For instance, search and alert radar can provide information for searching and rescuing, and meteorological radar can be used to alert and forecast the medium and small-scale weather systems (such as typhoons and storm clouds). Navigation radar can be used for navigation and positioning for aircraft, ships and other transportation vehicles. Healthy and stable radar equipment can improve the life quality of people and provide a guarantee for the safe operation of all types of vehicles. Therefore, it is indispensable to effectively evaluate and monitor the operation status of the radar equipment. Traditional radar equipment supporting methods include Breakdown Maintenance, Regular maintenance, On-condition Maintenance, etc. However, with the development of science and technology, these methods cannot meet the maintenance requirements of modern radar equipment, and present many shortcomings, such as weak ability of fault diagnosis, weak prediction of failure, rapid increase of protection costs, etc. [1]. An effective way to solve these problems is to establish an indicator system reasonably during the operation of the radar. The indicator system can reflect the operating status of the system. Then the health status of the radar system is evaluated in real time, and then the choice of maintenance decision is made, which is of great significance for improving the reliability and operational safety of the radar system.

Since radar systems are widely utilized in industrial applications [2] [3], there are quite a few studies on real-time health assessment of specific radar systems. Recently, most researches focus on monitoring and evaluating the health status of an equipment. Monitoring and managing the health of the equipment is also known as the Prognostics and Health Management (PHM). Terrissa LS, et al. indicated that PHM can offer significant benefits for maintenance so that it can predict the future behavior of a system as well as remain useful life in the manufacturing industry “industry 4.0” [4]. Aiming at monitoring of a product, especially for a complex equipment working in a harsh environment, Tao F, et al. put forward Digital Twin (DT), an emerging technology to achieve physical–virtual convergence for improving the accuracy and efficiency of PHM [5]. Ghiasi M, et al. used the means of minimizing the total system for improving the reliability of power network, and indicated that the main factors for evaluating the health status of an equipment include mean time between failure, mean time to repair, availability, system average interruption frequency index, system average interruption duration index, consumer average interruption duration index, average service availability index, and average service unavailability index, etc. [6]. Nevertheless, all the modelling and simulation of their work are implemented in the Electrical Transient Analyzer Program (ETAP) software. Yuan NQ, et al. [7] illustrated that vibration and acoustic signals collected from machine have been extensively and effectively applied in the health assessment field. A Deep Fast Random Forest (DFRF) fusion technique for machine running condition analysis was proposed. By using the wavelet packet transform (WPT), fault-sensitive statistical parameters of the vibration and acoustic signals are extracted firstly. Then two Deep Belief Networks (DBNs) were constructed to develop the deep representations of the WPT features. Finally, the Fast Random Forest (FRF) were utilized as an information fusion-based classification tool to fuse two kinds of deep features. Guo LJ, *et al*. integrated the power grid, equipment as well as environment data and the SVM method as the main algorithm to evaluate the risk of the main transformer [8]. Abushik GV, et al. firstly gave out the various limit states criteria of safety factors, and then assessed the remaining service life of turbine rotors only according to the various limit states criteria [9]. Liao WZ, et al. combined Simulated Annealing (SA) algorithm and Expectation Maximization (EM) algorithm together for machinery diagnosis, and the computation results illustrated the high efficiency and accuracy of the proposed method, which can help machinery diagnosis in practical situations. In addition, SA algorithm has strong ability of global convergence for optimizing the process of parameters estimation [10], which optimization process also gives us the inspiration of optimization.

Summarizing the used methods and techniques in the above mentioned literature, there are still some shortcomings in this research field. As observed from [11] [12], many researches mostly evaluate radar operational effectiveness and support capabilities, and its index system is mainly based on investigation and expert consultation, which is lack of real-time operational status data of the system. They cannot accurately judge the real-time health status of the system. Moreover, many researches study the radar evaluation index system, and Analytic Hierarchy Process (AHP) is always used to evaluate the health status of the radar in the evaluation process [13]. Although Fuzzy Comprehensive Evaluation considers the ambiguity between the evaluation indicators, the result is a certain state. If the system is at the edge of state, it is much possible to cause more serious losses for slow arrangements of maintenance strategy. Furthermore, in the processing method, many scholars always use Gray Clustering, AHP, Fuzzy Comprehensive Evaluation (FCE), other classical methods, and combinatorial methods to evaluate the equipment status.

To overcome the shortcomings of the above methods, this paper first investigates the technique of status recognition. We focus on establishing an evaluation index of radar equipment that including score index, potential condition and maintenance strategy and methods, etc., and most of the time the score is evaluated by specialists and life experience with strong subjectivity. However, the modern radar equipment has the interactions between different subsystems, and it is hard to reflect the real health condition of radar equipment based on the specialist scoring and life experience. In order to solve the evaluation problem, we first combine the subjective AHP method with objective Entropy weight method to comprehensively evaluate the value of the health condition. Then we seek the recognition methods for estimating the status of equipment when considering the actual issues that the existing training sample data are too limited. It is observed that Support Vector Machine Regression (SVR) model proposed for the classical binary classification provides good performance in the functional regression domain of small sample condition. Hereby we choose SVR model as the basic output model for estimating the health condition of the radar system. Note that Gauss Radial Basis Function (GRBF), the kernel of SVR, has good generalization in the local scale. It is necessary to use a series of process of optimization algorithm to the parameter *g* and penalty factor *C* of SVR model. Yang YF, et al. used CS algorithm to optimize the parameters of LSSVM, obtained the optimization result and proved its feasibility of those means [14]. By comprehensive studying the means in the literature [8] and [10], we choose the penalty factor *C* and the parameter *g* of GRBF for SVR model. In addition, we combine the FCE method to SVR model for guaranteeing the recognition accuracy. Therefore, the combined FCE, CS algorithm and SVR model are proposed for health assessment of the radar system. The newly proposed method can introduce merits such as making use of small sample data for analysis, reducing the computational complexity effectively, improving the accuracy of state assessment, and so on. Moreover, it avoids the problem that the system is at the edge of the state.

## 2. Data fusion and establishment of evaluation index system

According to the National Military Standard “Quality Monitoring Requirements for General Radar Equipment”, the percentage system is used to score the radar status. Radar is divided into health, sub-health, attention, deterioration and morbidity, see Table 1 for more details. The purpose of establishing scoring index is to propose a reference for expediently evaluating the health status.

**Table 1:** Radar system status and scoring recommendation form.

Score	0 ~ 25	25 ~ 50	50 ~ 75	75 ~ 85	85 ~ 100
**Possible state**	Morbidity (S5)	Deterioration (S4)	Attention (S3)	Sub-health (S2)	Health (S1)
**Maintenance strategy**	Arrange maintenance immediately	Repair as soon as possible	Priority arrangement	Delay planning	Delay

### 2.1 Data fusion

Due to the constraints of test conditions and test costs, the available test data are usually not enough, and the sample size of the health status assessment is very small. The data of health status assessment mainly include the data of on-line monitoring, off-line monitoring and radar technical indicators. The main information source for on-line monitoring is Built-in-test (BIT) system [15]. Off-line monitoring obtains maintenance data in the event of radar failure through portable maintenance equipment. The technical index of radar system is the index reflecting whether the radar can complete the specified function in the course of radar mission, including the operating range and sidelobe level.

The health assessment of the radar requires the fusion of the two types of monitoring data (on-line and off-line). In order to eliminate the influence of subjective scoring by experts, the Weighted method is first used to fuse two kinds of data. By setting on-line monitoring data as *x*_1_, and off-line monitoring data as *x*_2_, one can derive the result of data fusion as

$$\bar{x}=x_{1}\omega_{1}+x_{2}\omega_{2},\omega_{1}+\omega_{2}=1$$ (1)

where *ω*_1_, *ω*_2_ are the weights of on-line monitoring data, off-line monitoring data, respectively.

### 2.2 Establishment of evaluation index system

**Fig 1:**
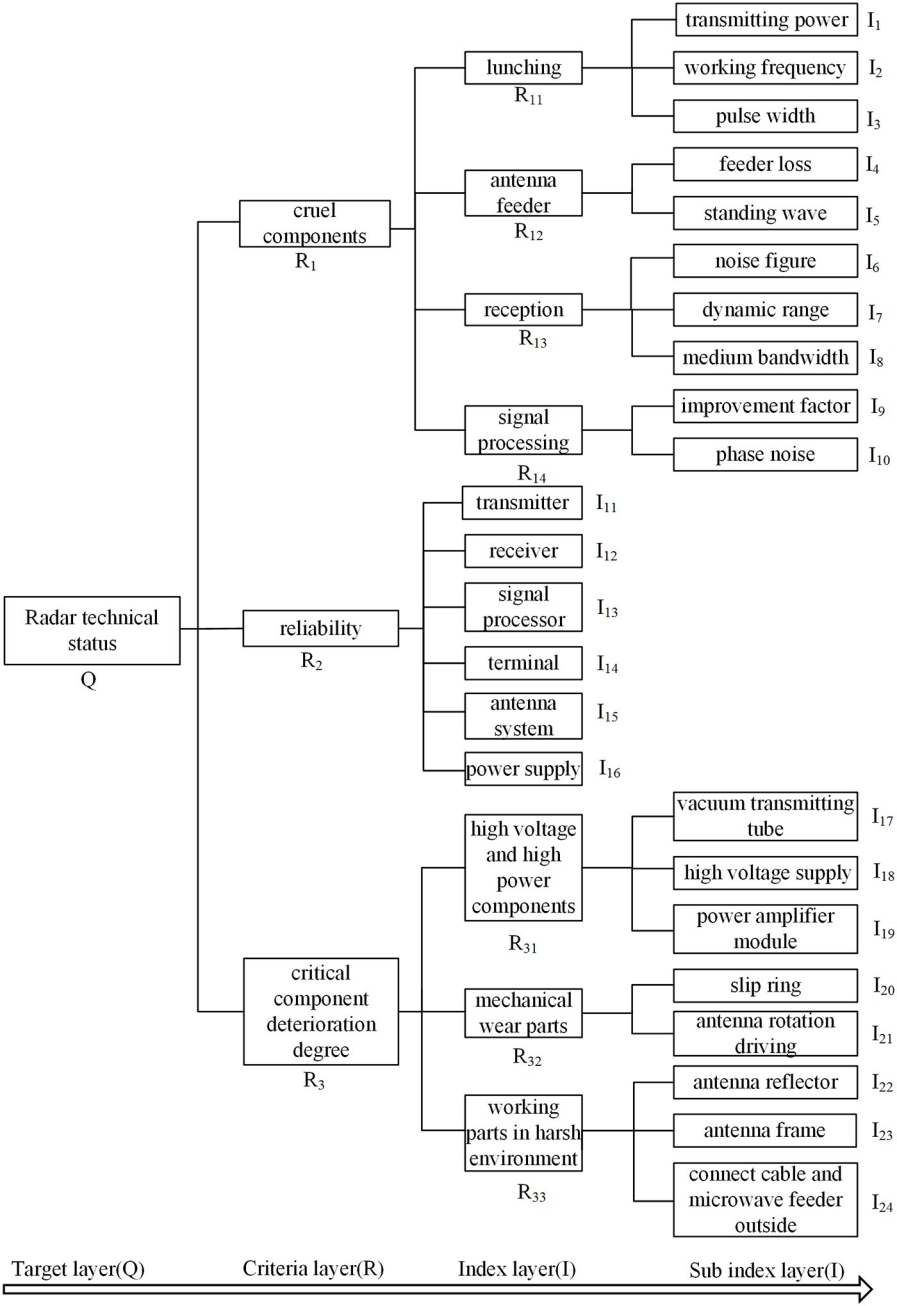
Status evaluation index system of the radar system.

The main performance index and the stability of the system can reflect the status of the radar system directly. We can get more accurate values of radar main performance index by testing. The index reflect the operation status of key components [16] (i. e. key and important functional equipment or components) of the system. System working stability is the ability of every subsystem to jointly perform the specified functions under specified conditions and time. According to the principle of establishing evaluation index system of hierarchical structure [17], a perfect evaluation index system is established in this paper, which is shown in Fig 1.

Due to the diversity of status assessment indicators and dimensions, measuring the merits of each evaluation index and unifying dimension are the primary problems of health assessment. Referring to literature [18], relative deterioration degree is used to express the degree of deviation of evaluation index from normal state, and to deal with the data after data fusion.

## 3. Health status evaluation based on Fuzzy Comprehensive Evaluation

### 3.1 Weight determination by combined weighting approach

Health status evaluation of radar system is a multi-attribute evaluation process. The weight of the index should reflect both the importance of the index and the information quantity of the index. Indicator weights obtained by a single method have one-sidedness. However, the weight gained by combined weighting approach can compensate for this shortcoming. Therefore, this section combines the feature of subjective and objective weighting methods. The combination of entropy weight method [19] [20] and AHP method [21] is used to determine the weight of evaluation index of the radar system.

Letting *W*_*A*_ and *W*_*B*_ represent the weights calculated by AHP and the Entropy Weight Method, respectively, and the linear Weighting method is used to calculate the final weight value

$$W=\theta W_{A}+(1-\theta )W_{B}$$ (2)

where *θ* is the ratio coefficient of subjective weight. The rationality of weighting coefficient is related to the correct application of the combined weighting method. As a link carrier between the two methods, it is necessary to reflect the preference characteristics of the evaluation subject and to express the consistency of different methods. In this paper, the difference coefficient method borrowed from [22] is adopted to calculate these two weighting coefficients

$$\theta =\frac{n}{n-1}T_{s}$$ (3)

$$T_{S}\text{=}\frac{2}{n}(1P_{1}+2P_{2}+\cdots nP_{n})-\frac{n+1}{n}$$ (4)

where *n* is the number of assessment indicators, *Ts* is the coefficient of variation between components in *W*_*A*_. *P*_1_, *P*_2_, ⋯, *P*_*n*_ represent the result of arranging the components of *W*_*A*_ in order of magnitude.

### 3.2 Health status evaluation based on Fuzzy Comprehensive Evaluation

**Fig 2:**
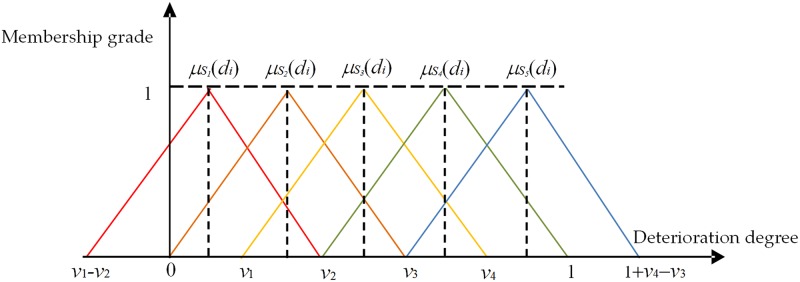
Subordinate function family of deterioration degree *d_i_* of evaluation index.

Combining with the evaluation index system in Fig 1, the Fuzzy Comprehensive Evaluation method [23] is used to evaluate the health status of multi-level and multi-factor radar systems. The main steps can be summarized as:

**Step 1**: Determining the set of evaluation factors *U* = (*u*_1_, *u*_2_, ⋯, *u*_*n*_).
**Step 2**: Determining the evaluation set V according to the health status of the radar system in Section 1.
**Step 3**: Determining the weight set, the combined weighting method is used to determine the weight of every influencing factor as *W* = (*w*_1_, *w*_2_, ⋯, *w*_*n*_).
**Step 4**: Determining the comprehensive evaluation matrix, constructing the triangle membership function as shown in Fig 2.
  Calculating the degree of index deterioration corresponds to the membership of every state, the fuzzy relation matrix of inferiority degree is derived as
  $$U=\{\begin{array}{ccccc} \mu_{s1}(d_{1}) & \mu_{s2}(d_{1}) & \mu_{s3}(d_{1}) & \mu_{s4}(d_{1}) & \mu_{s5}(d_{1})\\ \mu_{s1}(d_{2}) & \mu_{s2}(d_{2}) & \mu_{s3}(d_{2}) & \mu_{s4}(d_{2}) & \mu_{s5}(d_{2})\\ \vdots & \vdots & \vdots & \vdots & \vdots \\ \mu_{s1}(d_{n}) & \mu_{s2}(d_{n}) & \mu_{s3}(d_{n}) & \mu_{s4}(d_{n}) & \mu_{s5}(d_{n}) \end{array}$$ (5)
**Step 5**: Getting the status comment of radar through fuzzy comprehensive evaluation operation.
  $$B=W\times U,b_{j}=\sum_{i=1}^{n}w_{i}\mu_{sj}(d_{i})(j=1,2,\cdots ,5)$$ (6)
**Step 6**: Choosing the maximum subordination principle [24] to determine the final judgment result.

## 4. Evaluation model based on Fuzzy Comprehensive CS-SVR

Due to the result of evaluation is a certain state, if the system is at the edge of state, it will lead to more serious losses for slow arrangements of maintenance strategy. However, SVR can solve the learning problem of small samples of equipment data and the uncertainty of evaluation results. Therefore, the combination of Fuzzy Comprehensive Evaluation and SVR is used to evaluate the health status of the system. The prediction effect of SVR is not good because the parameters are artificially chosen [25]. In order to further improve the prediction accuracy, CS algorithm is used to optimize the parameters (the penalty factor *C* and the parameter *g* of GRBF) of SVR, and to optimize the performance of the SVR model correspondingly. Similar to the classical algorithms such as Particle Swarm Optimization (PSO) and genetic algorithm (GA), CS algorithm introduces the biological evolution theory. In addition, because the step size of the Levi flight satisfies the stable heavy-tailed distribution, the search result of CS is more effective. The parameters of CS algorithm are less than that of PSO and GA. Thus, the convergence of CS algorithm is faster than others. Comparing with other state-of-the art meta-heuristic algorithms (such as the Bat Algorithm, BA), the CS algorithm is proposed earlier (2009), and the application is less. However, it can be optimal within the global scope of specific conditions. It should be mentioned that the result obtained by CS algorithm optimizing the SVR model is fixed in the fuzzy boundary. We also improve the model (i. e. Fuzzy Comprehensive CS-SVR) for jumping out of fuzzy boundary (the status is at the edge of the state).

### 4.1 SVR model

In recent years, SVR [26] [27] has found wide applications in various problems of classification and regression. The basic idea of SVR is described as: transforming input vectors into high-dimensional space by non-linear transformation, and establishing regression functions in high-dimensional space by using the principle of structural risk minimization [28].

f(x)=w·Φ(x)+b (7)

where *x* ∈ *R*^*n*^ represents the input vector, *w* ∈ *R*^*n*^ represents the weight vector, while *b* ∈ *R* is the deviation.

In order to get *w* and *b*, the principle of structural risk minimization is adopted to transform the original problem to

$$\begin{array}{l} \text{min}\left[\frac{1}{2}{\left\|\omega \right\|}^{2}+C\sum_{i=1}^{l}\left(\xi_{i}+\xi_{i}{}^{*}\right)\right]\\ \text{s}.\text{t}.\;\;y_{i}-\omega_{i}-b\leq \varepsilon +\xi_{i}\\ \;\;\;\;\;\;\;\omega_{i}+b-y_{i}\leq \varepsilon +\xi_{i}{}^{*}\\ \;\;\;\;\;\;\;\;\;\;\;\;\;\xi_{i},\xi_{i}{}^{*}\geq 0 \end{array}$$ (8)

In the above given formula, ‖ω‖^2^ is complexity description of function *f*, *C* > 0 is a penalty coefficient, $\xi_{i},\;\xi_{i}{}^{*}$ are slack variables, *ε* is insensitive loss function. In order to solve the above convex optimization problem, Lagrange multiplier $\alpha_{i}\left(\alpha_{i}{}^{*}\geq 0\right)$ is introduced to construct Lagrangian function. The function is minimized by derivation and then transformed into its dual form to obtain the support vector non-linear regression function.

$$f(x)=\sum_{i=1}^{n}\left(\alpha_{i}^{*}-\alpha_{i}\right)K\left(x,x_{i}\right)+b$$ (9)

According to the Mercer condition, the Gauss radial basis function [29] is selected as the kernel function

$$K(x,x_{i})=\text{exp}\left\{-\frac{{\left\|x-x_{i}\right\|}^{2}}{g^{2}}\right\}$$ (10)

### 4.2 CS-SVR model

**Fig 3:**
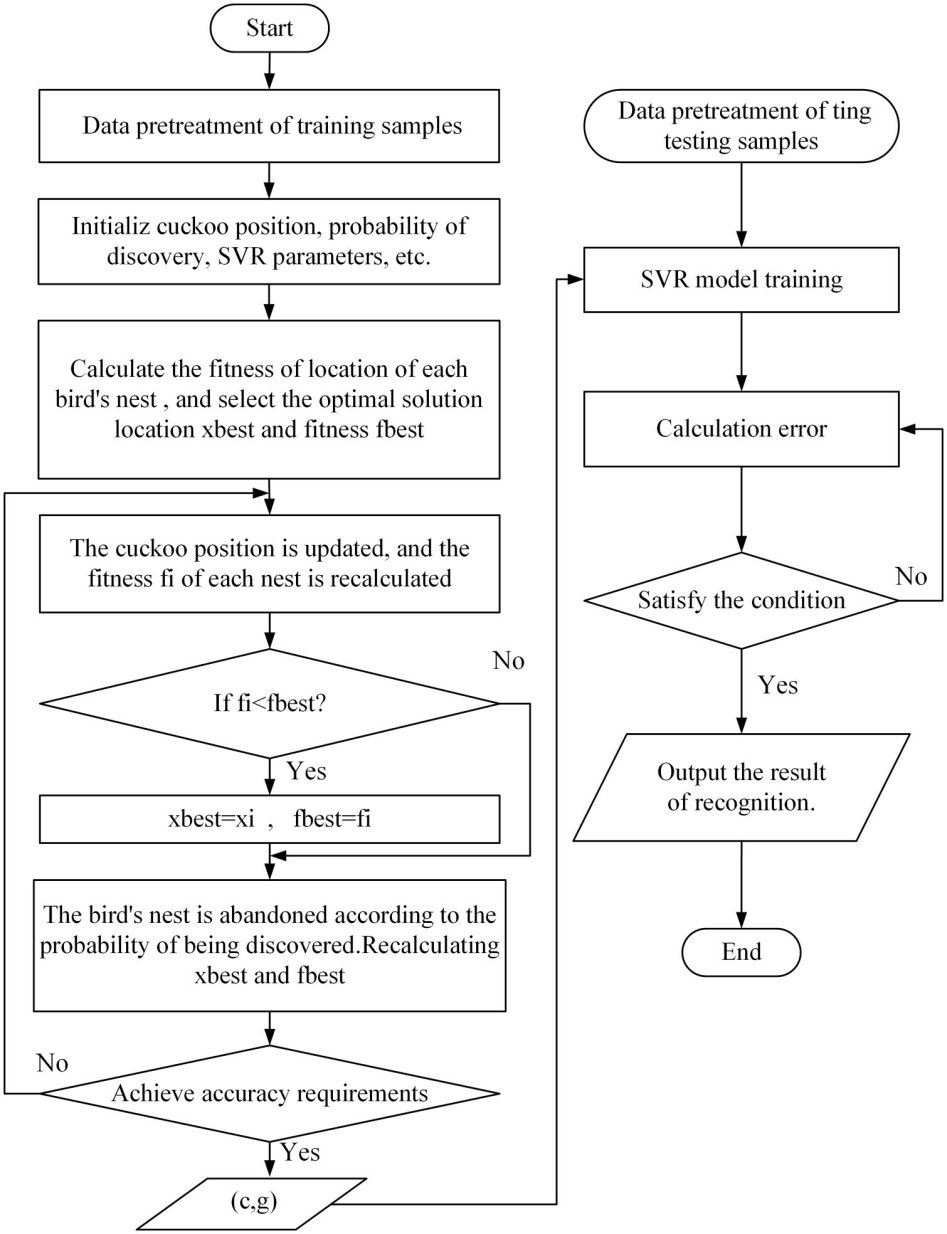
Flow chart of CS optimizing SVR model.

Penalty factor *C* and kernel function parameter mainly determine the generalization performance of SVR [30]. Using CS to optimize the parameters can optimize the performance of SVR model and avoid blind selection of parameters caused by artificial selection. The procedure of CS optimizing SVR model parameters are shown in Fig 3.

### 4.3 Health evaluation model based on fuzzy evaluation combined with CS-SVR

The method of Fuzzy Comprehensive Evaluation considers various state information of radar system comprehensively. The result shows the health status of the radar system. However, different methods may get different results in the final processing of the vector B of fuzzy operation, which may lead to judgment failure. SVR demonstrates the characteristics of being able to solve non-linear and small sample problems. Therefore, SVR is applied to deal with the fuzzy operation vectors to establish the model of Fuzzy Comprehensive CS-SVR (FCCS-SVR), and CS algorithm is used to optimize the parameters of SVR model. Specific steps are given as follows:

**Step 1** to **Step 6** are the same as those in section 3. 2.
**Step 7**: Training CS-SVR modeling, the status comment B of radar is divided into the set of training sample and the set of validation sample. Training sample is sent to CS-SVR model to obtain the optimal parameters. Testing sample is used to validate the accuracy of parameters.
**Step 8**: Using the CS-SVR model to evaluate the health status of radar system.

The performance index of CS-SVR model include MSE and regression coefficient *R*. The model fitness is the best when MSE is the smallest and *R* is the largest.

## 5. Case analysis of health status evaluation

The case analysis is mainly based on a certain type of radar. According to the above research, its health status is evaluated.

### 5.1 Performance analysis of Fuzzy Comprehensive CS-SVR model

In order to certify the validity and superiority of chosen algorithms and improved methods, we put forward the means of comparing CS-SVR model and other meta-heuristic algorithm optimization models with SVR. This paper has collected 60 groups of radar data, including the processed evaluation index data and the health status data of radar equipment scored by experts. The health status data rated by experts is taken as the actual value. In order to get the set of status comment, 60 groups of processed evaluation index data are used for fuzzy evaluation. Among them, 30 groups are chosen as training sample and the remaining 30 groups are chosen as testing sample. The set of status comments is used as SVR input, and the health status data scored by experts are used as output to train and validate SVR model. If the test sample set has good regression fitting effect, the model is proved to be able to evaluate radar health status correctly. The set is used as SVR input, and the health status data of radar equipment scored by experts is used as output to train and validate the support vector machine model.

The weight of each index is determined by the method of combined weighting, which is according to the criterion given in Table 1, the result is shown in Table 2.

**Table 2:** Comprehensive weights of every index (part).

Criterion (weight)	Index(weight)	Code	Comprehensive weight
Technical index *R*_1_ (0.3423)	Lunching *R*_11_ (0.4952)	*I*_1_ (0.7245)	0.1228
		*I*_2_ (0.1056)	0.0179
		*I*_3_ (0.1699)	0.0288
	Antenna feeder *R*_12_ (0.1249)	*I*_4_ (0.2013)	0.0086
		*I*_5_ (0.7987)	0.0341
	Reception *R*_13_ (0.2120)	*I*_6_ (0.6398)	0.0464
		*I*_7_ (0.2230)	0.0169
		*I*_8_ (0.1372)	0.0100
	Signal processing *R*_14_ (0.1679)	*I*_9_ (0.6518)	0.0374
		*I*_10_ (0.3482)	0.0200

The observed values and corresponding degradation degrees of radar evaluation index are shown in Table 3.

**Table 3:** Observations of state evaluation index (part).

Index	Unit	Range	Observation	Inferiority degree
*I*_1_	kW	(180–220)	182	0. 15
*I*_2_	MHz	1210±20	1240	0. 03
*I*_3_	(edge)*μs*	≤0. 1	0. 09	0. 12
*I*_4_	dB	≤5. 6	6. 0	0. 14
*I*_5_	-	≤1. 4	1. 43	0. 16
*I*_6_	dB	(1. 5–3. 0)	2. 22	0. 08
⋮	⋮	⋮	⋮	⋮
*I*_24_	-	-	-	0. 12

The principal value region of status S1, S2, S3, S4 and S5 corresponding to every evaluation index determined by experts are shown in Table 4.

**Table 4:** The principal value region of every status.

Status	S1	S2	S3	S4	S5
Deterioration range	[0. 00, 0. 05]	[0. 05, 0. 15]	[0. 15, 0. 30]	[0. 30, 0. 45]	[0. 45, 1. 00]

**Fig 4:**
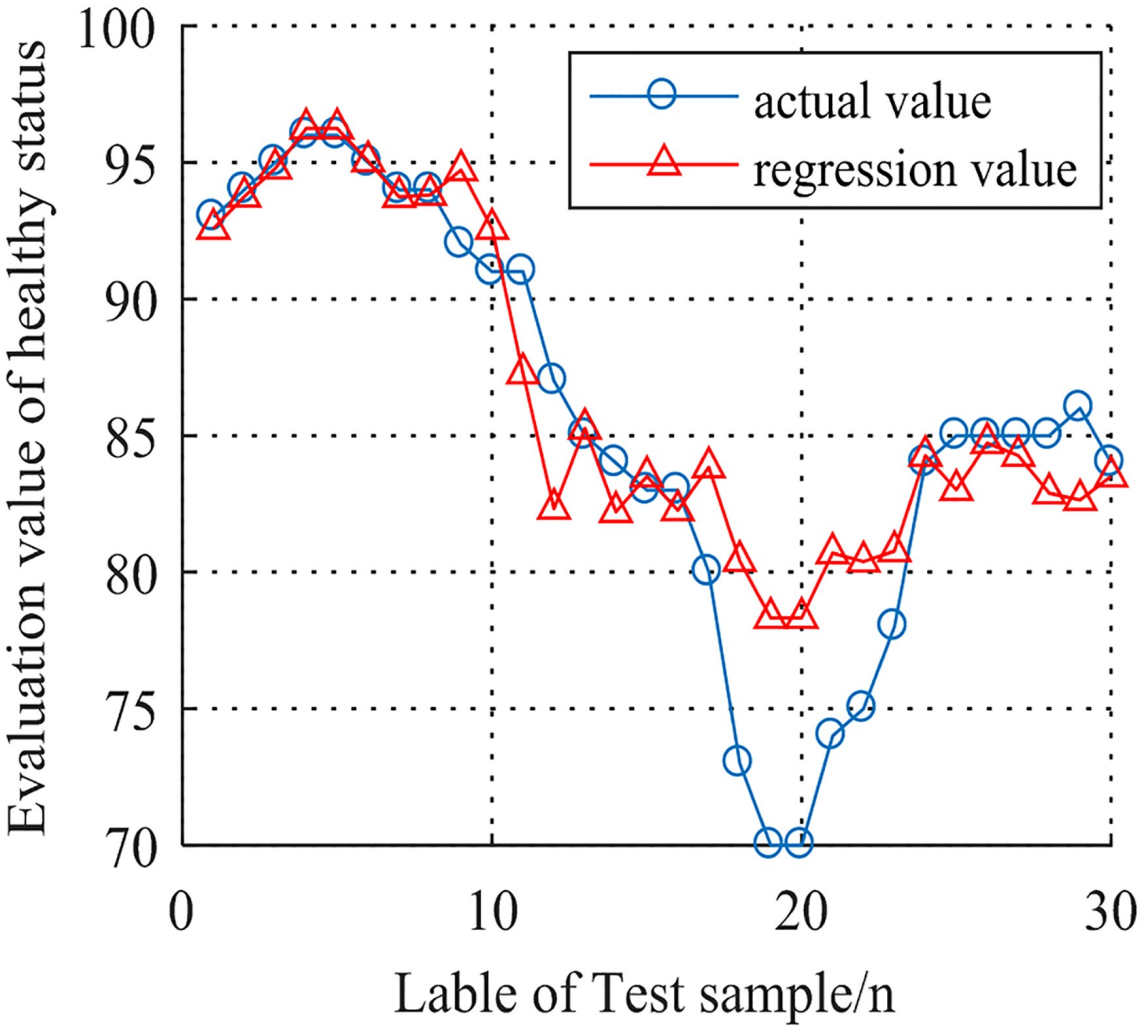
Comparison of actual value of validation sample with SVR regression value of stochastic optimization.

According to the data in Table 4, the triangular membership function is constructed, and the membership function is used to calculate the membership degree of the state corresponding to the deterioration degree of every index by the membership function. Based on the 5 value ranges of radar system status, the comprehensive value of MSE and *R* is set as the fitness in the searching process. The regression fitting effect of the model of stochastic optimized SVR is shown in Fig 4. The stochastic optimization is automatically operated by SVR tools of MATLAB. In addition, the iteration termination of stochastic optimization is 200, and the population number is 20.

**Fig 5:**
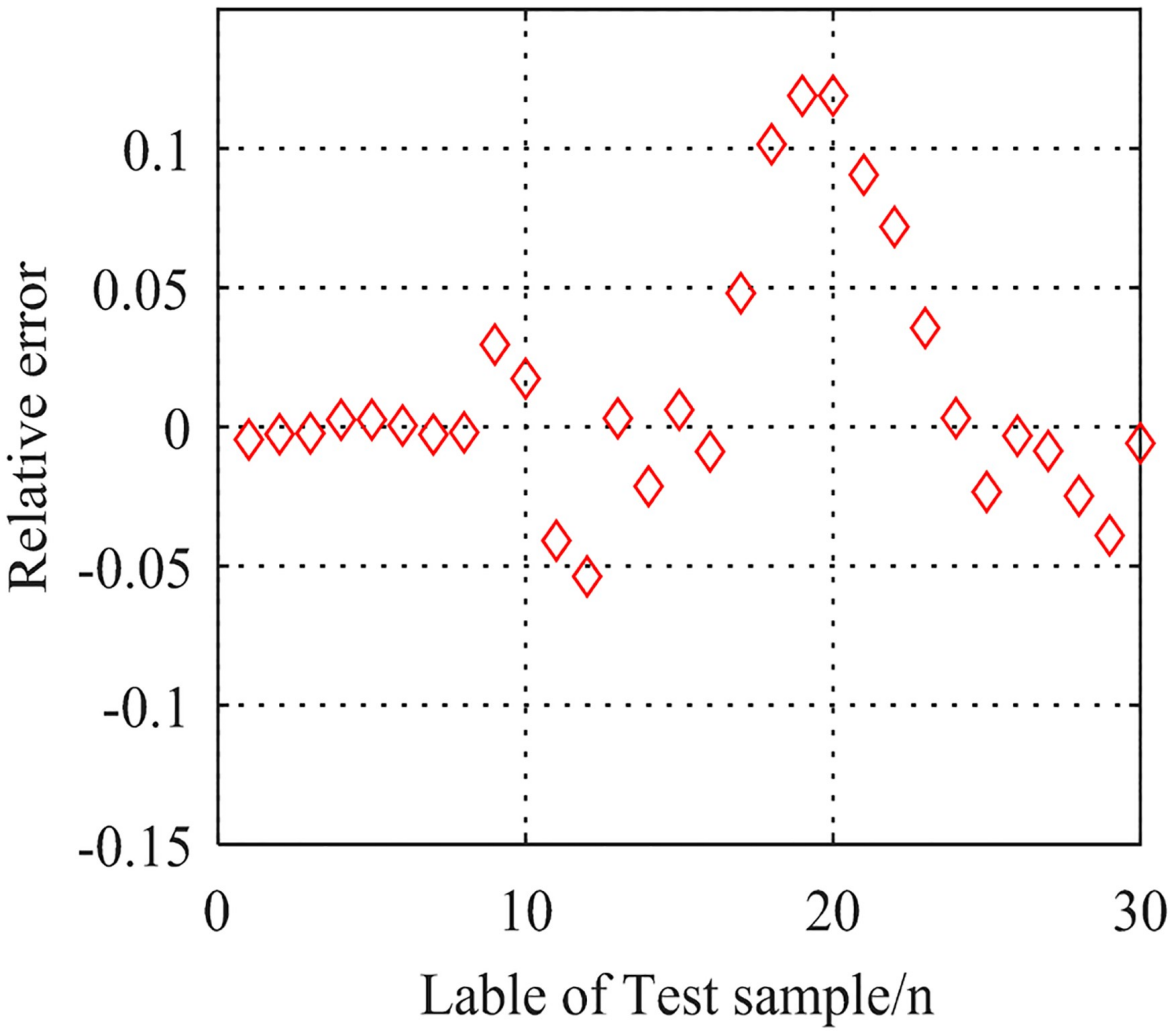
Relative error of the SVR regression of stochastic optimization.

As shown in Fig 4, the penalty factor C = 5.34099, the parameter of Gauss kernel function *g* = 1.0921. The regression coefficient *R* = 0.830575 is obtained by simulation calculation. The relative error is shown in Fig 5. From Figs 4 and 5, one can see that the fitness of regression of stochastic optimization is not good and the relative error is relatively high.

**Fig 6:**
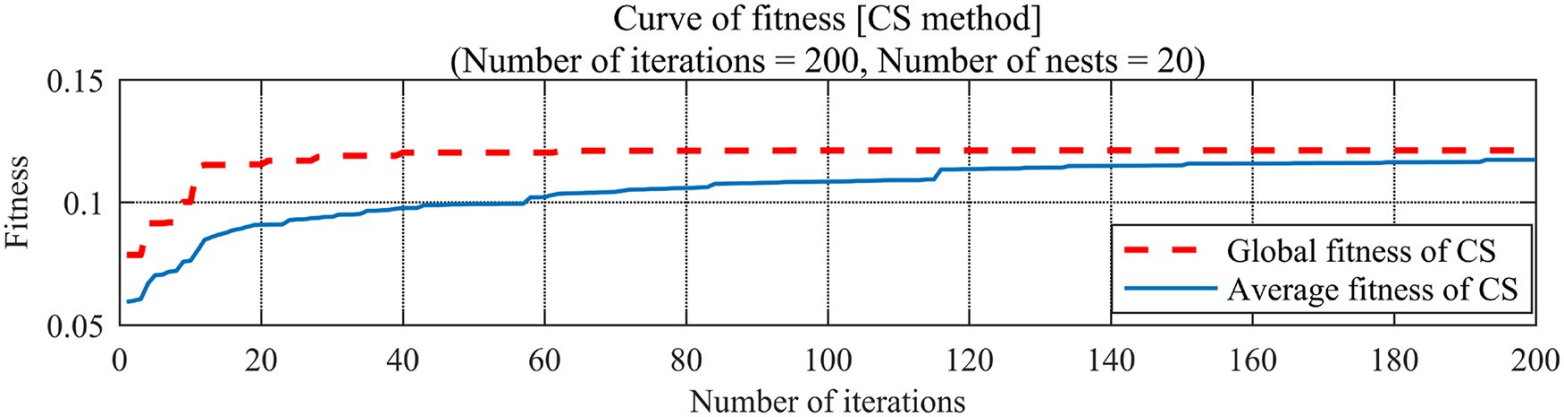
The fitness curve of CS-SVR (Termination of iteration = 200, Number of nest Nest = 20).

The fitness curve of CS-SVR is shown in Fig 6. When MSE = 0.0047, the fitness of CS-SVR model is the best, the optimal penalty factor *C* = 25.6030, and the optimal parameter of Gauss Kernel Function *g* = 6.3621.

**Fig 7:**
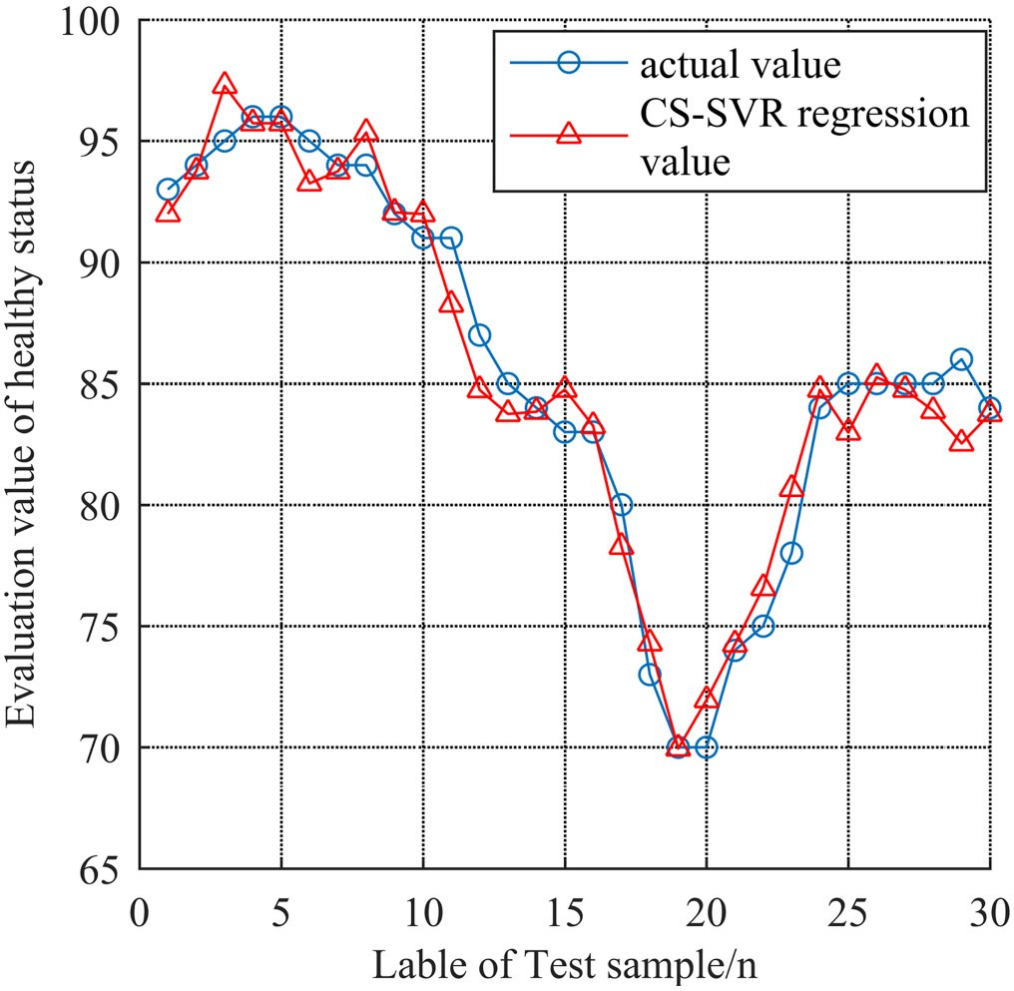
Comparison of the actual value of validation samples with the regression of CS-SVR.

**Fig 8:**
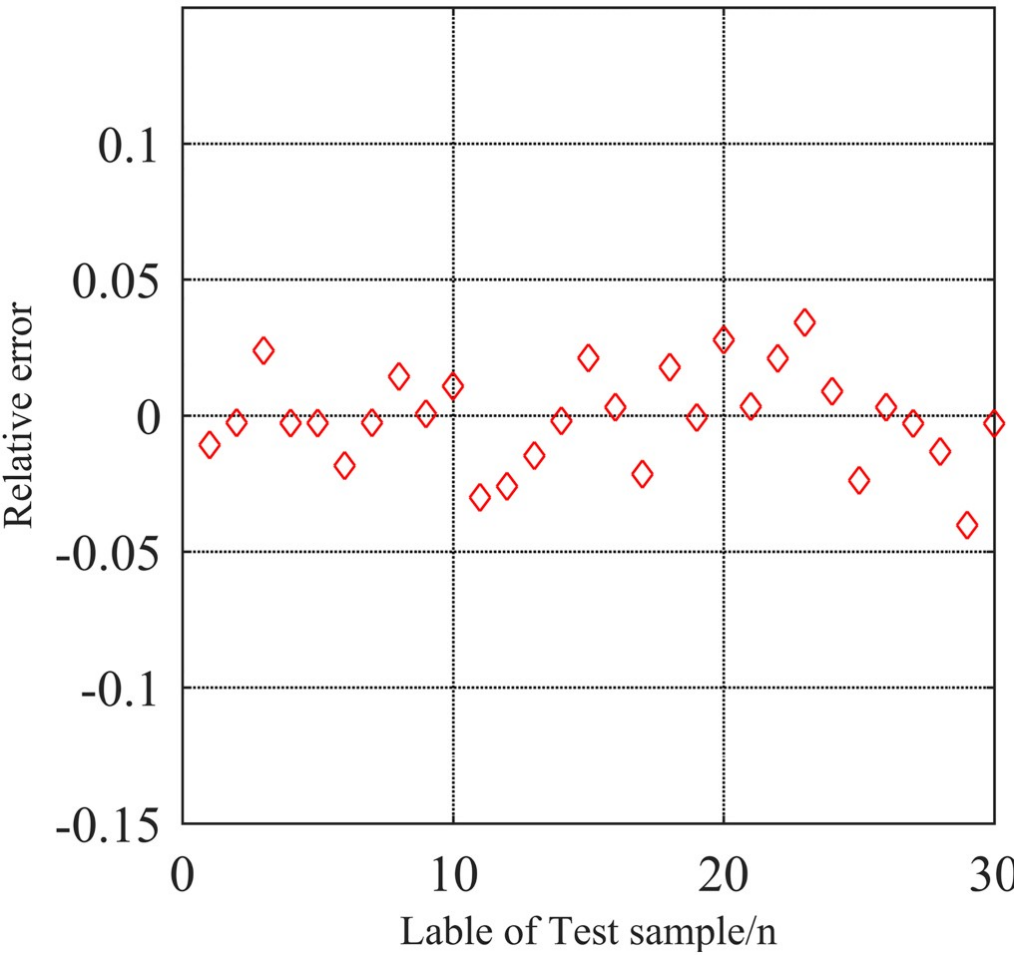
Relative error diagram of CS-SVR regression.

The regression prediction of CS-SVR is shown in Fig 7. The regression coefficient *R* = 0.9632 by simulation, and the relative error *E* is less than 0.05, which is presented in Fig 8. As one can see from Figs 7 and 8, The CS-SVR model provides a higher regression coefficient, smaller relative error, higher fitting degree and higher evaluation accuracy than the model of stochastically optimized SVR, which illustrates the merits of the model of CS-SVR.

**Fig 9:**
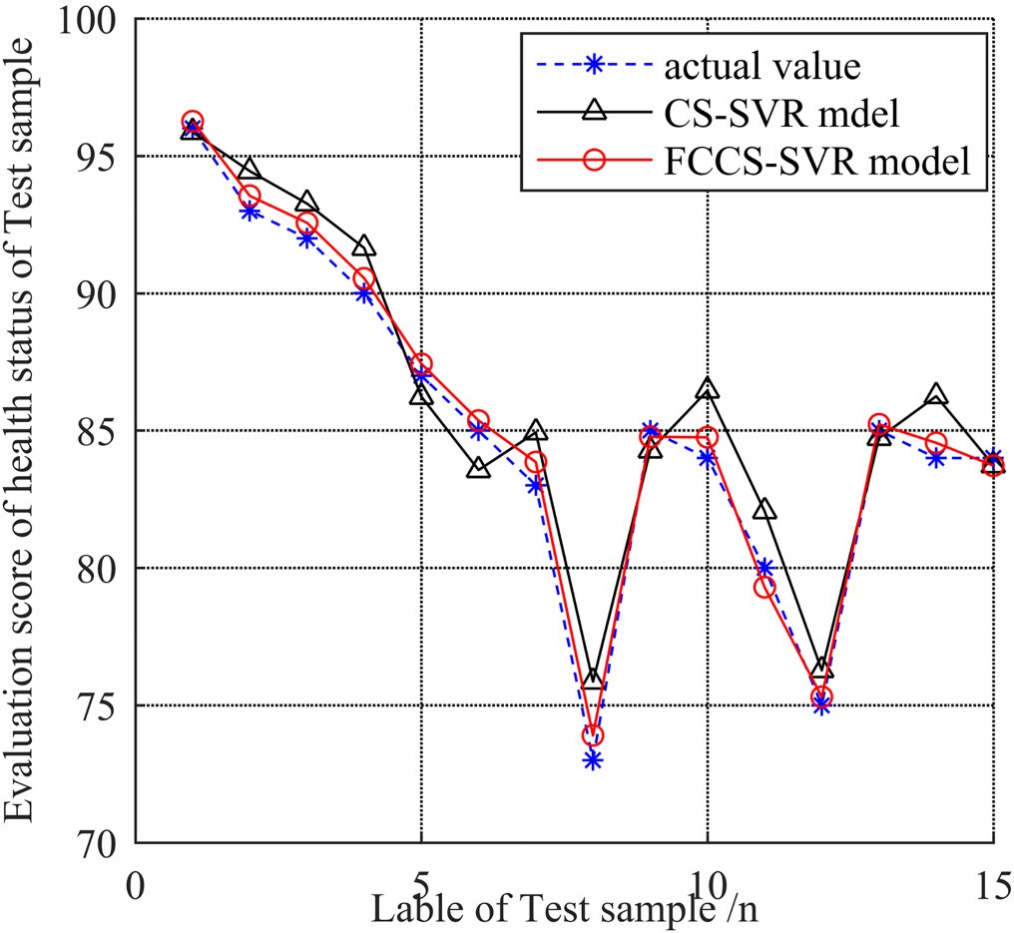
Comparison of the evaluation results of FCCS-SVR model and CS-SVR model (Termination of iteration = 200, Number of nest Nest = 20).

The results of Figs 7 and 8 demonstrate that the CS-SVR model is valid. In order to illustrate the superiority of the model, the same training set and verification set are directly input into CS-SVR model without fuzzy evaluation. Fifteen groups of data are selected to be evaluated by the model of FCCS-SVR and CS-SVR model. The results are shown in Fig 9.

**Fig 10:**
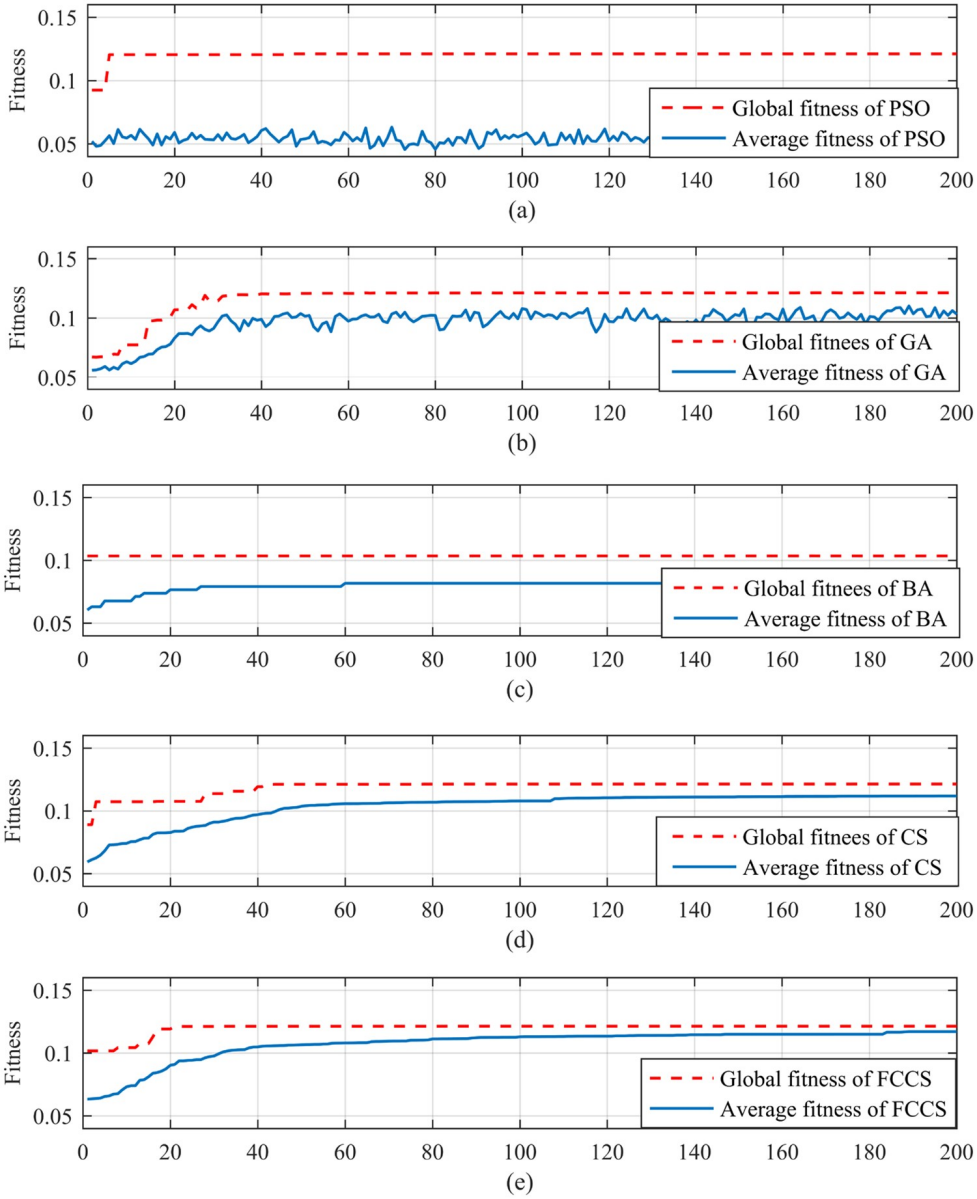
Comparison of the convergence of PSO-SVR, GA-SVR, BA-SVR, CS-SVR and FCCS-SVR.

As one can see from Fig 9, the model of fuzzy evaluation combined with CS-SVR (FCCS-SVR) can provide more accurate assessment results. The 15 groups of evaluation values of FCCS-SVR are 95.8633, 94.4439, 93.2633, 91.6443, 86.2321, 83.5633, 84.9312, 75.8583, 84.2707, 86.4507, 82.0627, 76.2691, 84.3283, 86.2507 and 83.7323. In addition, in order to further illustrate the superiority of the CS algorithm when dealing with the health status evaluation problems of complexity equipment, the comparison of convergence from CS-SVR, FCCS-SVR and SVR models combined with other classical optimization algorithms such as Particle Swarm Optimization (PSO), genetic algorithm (GA), meta-heuristic algorithms, and Bat algorithm (BA) is presented in Fig 10. In addition, the computational time of PSO-SVR, GA-SVR, BA-SVR, CS-SVR and FCCS-SVR are 16.46s, 30.53 s, 21.87 s, 22.47 and 11.79 s, respectively.

**Fig 11:**
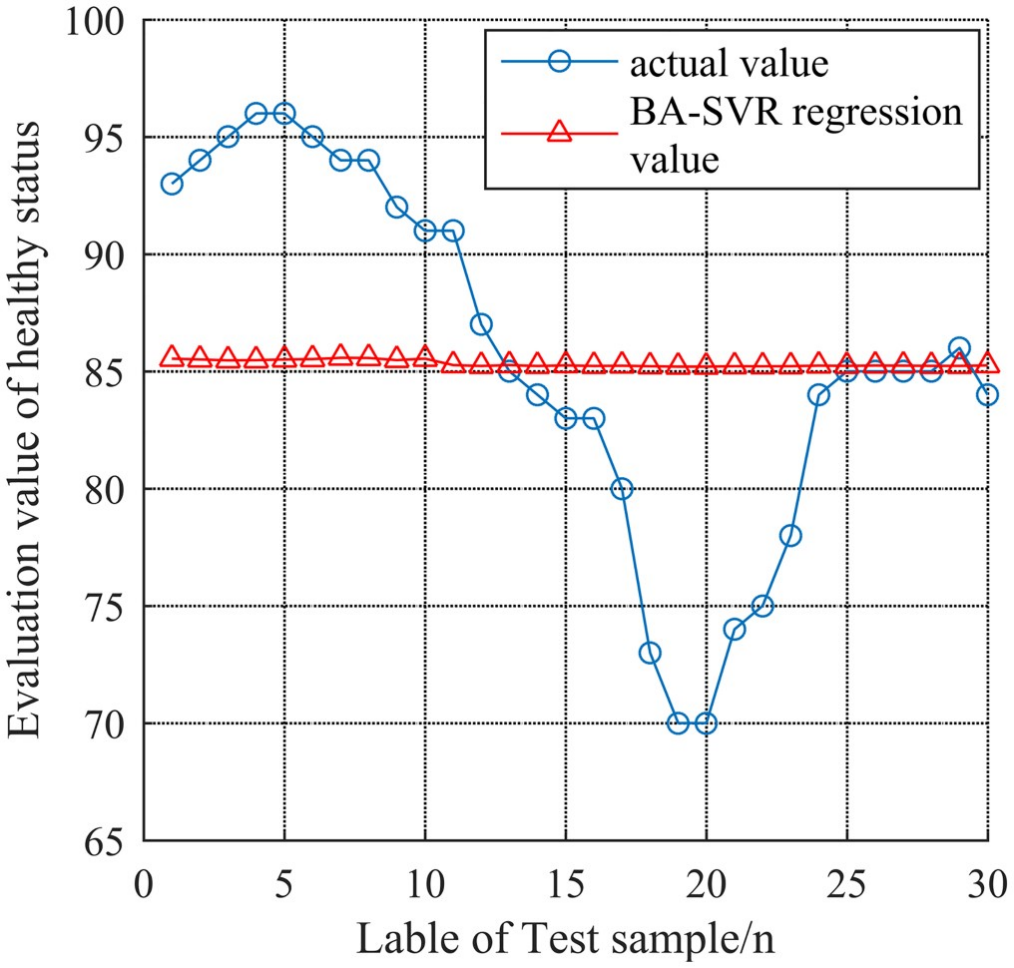
Evaluation result of BA-SVR.

In Fig 10, (a) is the convergence curve of PSO-SVR, (b) is the evaluation result of GA-SVR, (c) is the convergence curve of BA-SVR, (d) is the convergence curve of CS-SVR and (e) is the convergence curve of FCCS-SVR. According to the convergence curve of (e), the FCC-SVR model is more easier to achieve the global optimum when the status is at the edge of state. Particularly, as shown in Fig 11, the 30 groups of evaluation result of BA-SVR give out a high error compared with the actual one (Hereby, population number is 20, the iteration termination is 200).

Therefore, the method combines the advantages of Fuzzy Comprehensive Evaluation and SVR. It not only takes into account the fuzzy relationship of various factors, but also makes full use of SVR to solve the problem of the system at the edge of the state. The characteristics of small sample and strong generalization ability of SVR have been brought into full play, and it is a recommendable method.

### 5.2 Health status analysis of Fuzzy Comprehensive CS-SVR model

**Fig 12:**
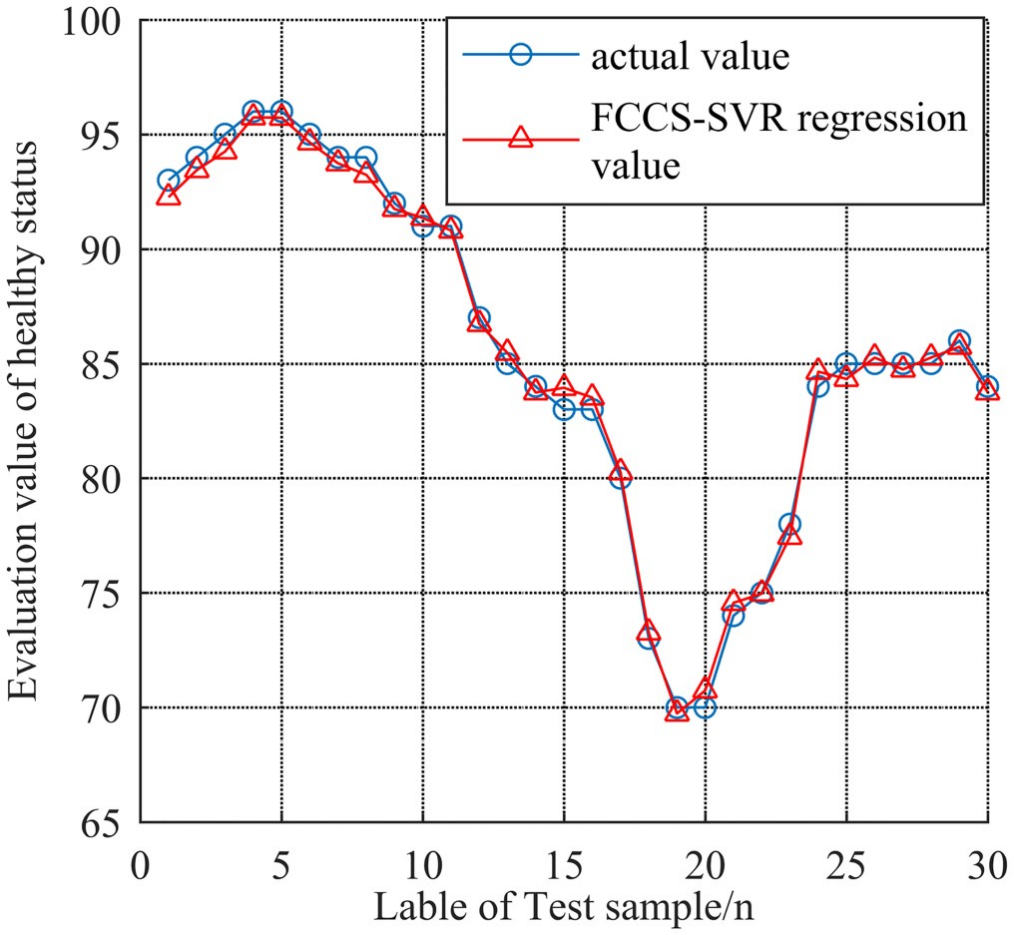
30 groups of evaluation value by using FCCS-SVR.

Based on the same index data and parameters obtained in section 5.1, the proposed FCCS-SVR model is used for evaluating the health status of radar system by 30 groups of data, which can be seen in Fig 12. The specific values are shown in Table 5.

**Table 5:** Evaluation values based on FCCS-SVR.

Label	Actual value	Evaluation result	Relative error
1	93	92.2585	-0.0080
2	94	93.4409	-0.0059
3	95	94.2499	-0.0079
4	96	95.7411	-0.0027
5	96	95.7275	-0.0028
6	95	94.6466	-0.0037
7	94	93.7382	-0.0028
8	94	93.2357	-0.0081
9	92	91.7426	-0.0028
10	91	91.3585	0.0039
11	91	90.8080	-0.0021
12	87	86.7286	-0.0031
13	85	85.4675	0.0055
14	84	83.7388	-0.0031
15	83	83.9431	0.0114
16	83	83.5202	0.0063
17	80	80.2631	0.0033
18	73	73.2613	0.0036
19	70	69.7371	-0.0038
20	70	70.7371	0.0105
21	74	74.5615	0.0076
22	75	74.9624	-0.0005
23	78	77.4392	-0.0072
24	84	84.6480	0.0077
25	85	84.3329	-0.0078
26	85	85.2592	0.0030
27	85	84.7480	-0.0030
28	85	85.2654	0.0030
29	86	85.7311	-0.0031
30	84	83.7431	-0.0031

When MSE is 0.0003, the optimum adaptability of FCCS-SVR is best, the optimal penalty factor *C* is 99.9991, and the optimal parameter *g* of GRBF is 144.8317.

In addition, Table 6 gives out the evaluation values from different models proposed before. The evaluation result of health status by FCCS-SVR is close to the actual result, which proves the validity and feasibility of that method. Meanwhile, a comparative analysis of the models shows that the larger the regression coefficient *R* is, the closer the evaluation result is to the actual value.

**Table 6:** Parameters and evaluation accuracy by different models.

Models	Training samples/group	Regression coefficient *R*	Penalty factor *C*	Parameter of GRBF *g*	Evaluation accuracy/%
PSO-SVR	30	0.9023	56.2374	19.2616	94.13
GA-SVR	30	0.9204	17.4895	8.2546	96.32
BA-SVR	30	0.9503	71.0592	153.7255	98.03
CS-SVR	30	0.9632	25.6030	6.3621	98.64
FCCS-SVR	30	0.9971	99.9991	144.8317	99.31

Based on all analysis above, the status comments are calculated in Table 7.

**Table 7:** Status comment.

Deterioration degree	Membership of each status				
	S1	S2	S3	S4	S5
*d*_1_	0	0. 75	0. 5714	0	0
*d*_2_	0. 96	0. 3	0	0	0
*d*_3_	0. 24	0. 9	0. 56	0	0
*d*_4_	0. 8	0. 8	0. 5143	0	0
*d*_5_	0	0. 7	0. 6286	0. 0167	0
*d*_6_	0. 56	0. 8	0. 1714	0	0
⋮	⋮	⋮	⋮	⋮	⋮
*d*_24_	0. 24	0. 9	0. 56	0	0

According to formula (6), the status comment *B* = (0.1544, 0.6283, 0.3005, 0.0832, 0) of radar is obtained. The status of radar system is evaluated as 75.8 points which through inputting the status comments into the trained FCCS-SVR model. The result shows that the equipment status is sub healthy, and the corresponding maintenance strategy is delayed or planned maintenance.

## 6. Conclusions

The radar system, which is a complex equipment with different sub-systems, has the characteristics of small and non-linear monitoring signal samples. In this paper, the scoring index is first established by the AHP method and the weighted method. On the basis of studying the characteristics of the radar system and many recognition models, we attempt to use different optimization algorithms including PSO, GA and newly proposed BA to optimize the parameters of SVR model. Compared with the actual values of that status, it reflects that those models exist high relative errors when the equipment status is at the edge of state. Due to the good performance of Levi flight satisfying the stable heavy-tailed distribution, the CS-SVR model is proposed. In order to further eliminate the fuzzy boundary problem, the Fuzzy Comprehensive Evaluation and SVR model are combined together to study the health status method of the radar system. The parameters of SVR model optimized by CS algorithm are simple to set, the computational complexity is reduced and the accuracy is also improved. Through comparing the evaluation values among PSO-SVR, GA-SVR, BA-SVR, CS-SVR and FCCS-SVR, the simulation results have illustrated that the FCCS-SVR model can provide high accuracy of 99.31%, which is better than other proposed models. In addition, the combined methods cost more time for evaluating the values of health status, but the evaluation accuracy is higher than that of single-algorithm one. The comprehensive value 75.8 points of the radar system is calculated out according to formula (6). All works have proved the validity and feasibility of PSO-SVR, GA-SVR, BA-SVR, CS-SVR and FCCS-SVR on the evaluation of status condition. Meanwhile, the FCCS-SVR provides a better performance under the edge of state environment after the case analysis. Finally, the status comments are put forward to provide technical support for decision-making of maintenance of the radar system. Due to time constraint, we have not used more meta-heuristic algorithms to optimize SVR model for evaluating health status and made maintenance strategy for each status. Hence, a further study of FCCS-SVR such as computational cost and maintenance strategy applied to the whole process of health evaluation will be considered.
